# Association of *CHD8* Gene Polymorphic Variants with the Clinical Phenotype of Autism Spectrum Disorder

**DOI:** 10.3390/jcm13237019

**Published:** 2024-11-21

**Authors:** Tomasz Iwanicki, Joanna Iwanicka, Anna Balcerzyk-Matić, Alicja Jarosz, Tomasz Nowak, Ewa Emich-Widera, Beata Kazek, Agnieszka Kapinos-Gorczyca, Maciej Kapinos, Katarzyna Gawron, Aleksandra Auguściak-Duma, Wirginia Likus, Paweł Niemiec

**Affiliations:** 1 Department of Biochemistry and Medical Genetics, School of Health Sciences in Katowice, Medical University of Silesia in Katowice, Medykow Street 18, 40-752 Katowice, Poland; jiwanicka@sum.edu.pl (J.I.); abalcerzyk@sum.edu.pl (A.B.-M.); alicja.jarosz@sum.edu.pl (A.J.); tnowak@sum.edu.pl (T.N.); pniemiec@sum.edu.pl (P.N.); 2 Department of Pediatric Neurology, Faculty of Medical Science in Katowice, Medical University of Silesia in Katowice, Medykow Street 16, 40-752 Katowice, Poland; eemich-widera@sum.edu.pl; 3 Child Development Support Center Persevere, Kępowa Street 56, 40-583 Katowice, Poland; beakazek@op.pl; 4 CZP Feniks, Daily Ward for Children and Adolescents, Młyńska Street 8, 44-100 Gliwice, Poland; 5 Department of Molecular Biology and Genetics, Faculty of Medical Sciences in Katowice, Medical University of Silesia in Katowice, Medykow Street 18, 40-752 Katowice, Poland; kgawron@sum.edu.pl (K.G.); aaugusciak@sum.edu.pl (A.A.-D.); 6 Department of Anatomy, Faculty of Health Sciences in Katowice, Medical University of Silesia in Katowice, Medykow Street 18, 40-752 Katowice, Poland; wlikus@sum.edu.pl

**Keywords:** *CHD8* gene, polymorphisms, autism spectrum disorder, muscle hypotonia, low birth weight

## Abstract

**Background**: The *CHD8* gene encodes chromodomain helicase DNA-binding protein 8 (CHD8), which is a transcriptional regulator involved in neuron development, myelination, and synaptogenesis. Some *CHD8* gene mutations lead to neurodevelopmental syndromes with core symptoms of autism. The aim of this study was to perform an analysis of the family-based association of *CHD8* gene polymorphisms with the occurrence and clinical phenotype of autism spectrum disorder (ASD). **Methods**: We analyzed 210 Caucasian children with ASD and their biological parents. The genotyping of specified polymorphisms, i.e., rs7148741, rs35057134, and rs10467770, was performed using TaqMan-PCR and compared with specific symptoms of ASD. **Results**: The G allele (rs7148741) was associated with muscle hypotonia as compared with the AA homozygotes. AA homozygosity (rs35057134) predisposed an individual to the use of an incubator, heart rate fluctuations, and the necessity of hospitalization. Moreover, the alleles and genotypes of this polymorphism were characterized by different Apgar scores and distributions. Additionally, CC homozygotes of rs10467770 were more often predisposed to the use of an incubator and hospitalization relative to T allele carriers. The average Apgar score was higher in TT homozygotes. **Conclusions**: Polymorphisms of the *CHD8* gene may determine specific clinical phenotypes of ASD.

## 1. Introduction

**Figure 1:**
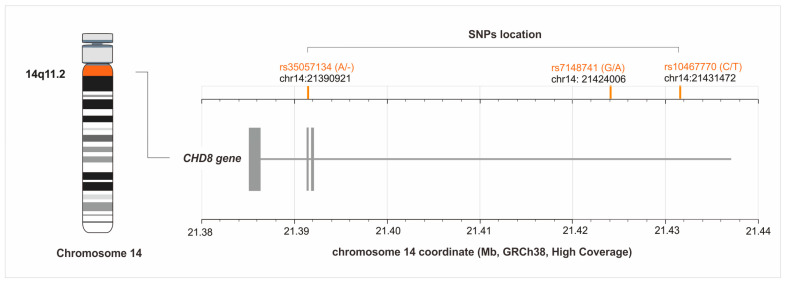
Location of the *CHD8* gene’s single nucleotide polymorphisms (SNPs) on chromosome 14 (GRCh38). The figure was created on the basis of data from LDmatrix Tool [12].

Autism spectrum disorder (ASD) includes a group of multifactorial neurodevelopmental disorders, diagnosed in 1 out of 68 (1.4%) individuals in the general population [1]. Studies performed in the last decade uncovered hundreds of genes that contribute to the occurrence of serious deficits in communication, social skills, and behavior, which are often experienced by ASD patients, highlighting the genetic heterogeneity of the disease [2]. The *CHD8* gene is located in the 14q11.2 region and encodes chromodomain helicase DNA-binding protein 8 (CHD8). This transcription regulator is expressed in almost all cell types and is involved in many cellular processes, including the cell cycle, cell adhesion, neuron development, myelination, and synaptogenesis [3]. The *CHD8* gene is a high-confidence risk factor for ASD and a genetic cause of a distinct neurodevelopmental syndrome with core symptoms of autism, macrocephaly, and facial dysmorphia (OMIM # 615032) [4]. The *CHD8* peptide acts as a chromatin regulator that binds to the promoters of actively transcribed genes through genome targeting mechanisms [5]. Therefore, any dysfunction of the *CHD8* protein may affect the expression of genes involved in neurodevelopmental pathways and the pathogenesis of ASD [6]; however, the specific pathomechanism linking these processes has not yet been fully understood. Recent data suggest that the mutations affecting the expression of *CHD8* (loss of function) may be related to autistic behaviors [7]. Recent studies conducted on the Brazilian Amazonian population [8] have demonstrated that the frequency of common polymorphic variants of *CHD8* may vary among populations, influencing the impact of SNPs on protein expression. The selection of the gene was based on an analysis by Satterstrom et al. [9], which indicated that the *CHD8* gene exhibited one of the lowest false-discovery rates in the association study. The selection of polymorphisms was based on available information in the literature. The rs10467770 polymorphism has been associated with Social Responsiveness Scale (SRS) T-scores and intelligence quotient (IQ) scores in the Japanese population [10]. The functional impact of this variant predicted by SNPeff was moderate [8]. In the case of rs7148741, a genome-wide association study showed strong association of this variant with working memory brain activation. This led the authors to assume that this variant may be relevant to the susceptibility of individuals to elements commonly affecting brain function. It is known that working memory may be impaired in individuals with ASD [11]; therefore, rs7148741 seemed interesting. We also considered the frequency of the minor allele, which was at least 20% (http://www.1000genomes.org; accessed on 13 September 2024), and analyzed the impact of SNPs on gene expression in tissues. Therefore, in the current study, we focused on the association analyses of the rs10467770, rs7148741, and rs35057134 (Figure 1) polymorphic variants of the *CHD8* gene with the clinical phenotype of ASD, considering, among other things, the motor and social abilities of patients.

## 2. Materials and Methods

This cohort study consisted of children with ASD and their biological parents and was conducted in accordance with STROBE guidelines. Three polymorphisms of the *CHD8* gene (two single nucleotide polymorphisms, rs10467770 and rs7148741, and one insertion/deletion polymorphism, rs35057134) were genotyped, and a transmission disequilibrium test (TDT) was performed to find a possible relationship between the chosen polymorphisms and ASD. An association analysis of genotypic variants of *CHD8* gene polymorphisms with the clinical phenotype of ASD was also performed.

### 2.1. Material

**Figure 2:**
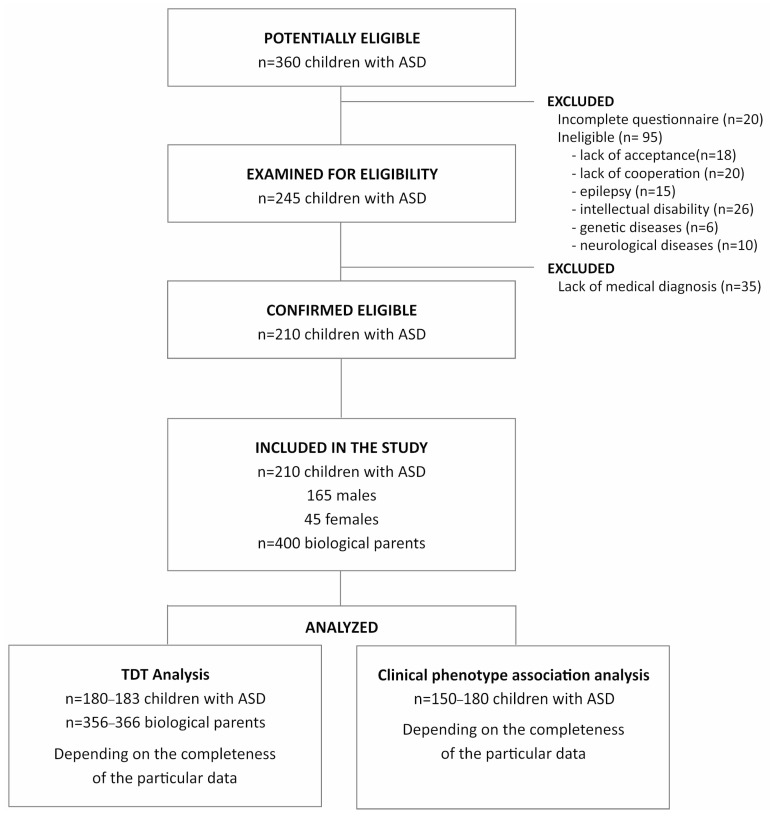
Flow chart of patient selection. Legend: ASD, autism spectrum disorder; TDT, transmission/disequilibrium test.

The study group comprised 210 children with idiopathic ASD and their biological parents. Patients enrolled included 165 males (78.64%) and 45 (21.36%) females (mean age 7.26 ± 2.72). All participants were Caucasians and inhabitants of Upper Silesia (southwestern region of Poland). Subjects were recruited between 2016 and 2019 at the Department of Pediatric Neurology (John Paul II Upper Silesian Child Health Centre, Katowice, Poland), the Child Development Support Center, and the Psychiatric Daily Ward for Children and Adolescents in Gliwice. The diagnosis was made by a psychiatrist specializing in autism spectrum disorders. The ADOS-2 (Autism Diagnosis Observation Schedule) protocol was used to confirm the diagnosis of ASD [13]. In addition to the diagnosis of autism, the inclusion criterion was age ranging from 3 to 12 years. Exclusion criteria were as follows: epilepsy, intellectual disability, and other genetic and neurological diseases. A flow chart of patient selection is presented in Figure 2.

This study was carried out in accordance with the Declaration of Helsinki and approved by the Bioethics Committee of the Medical University of Silesia in Katowice (Poland). Written informed consent was obtained from all subjects involved in the study. Informed consent was obtained from the legal parents of the children with ASD.

### 2.2. Genetic Analysis

Genomic DNA was isolated from peripheral blood leukocytes using the MasterPure™ genomic DNA purification kit (Epicentre Technologies, Maharashtra, India). The rs7148741, rs35057134, and rs10467770 polymorphisms of the *CHD8* gene were genotyped using a specific TaqMan^®^ Pre-designed SNP Genotyping Assay Kit (Applied Biosystems, Foster City, CA, USA). The PCR reaction and genotyping were performed according to the manufacturer’s instructions using a Roche LightCycler^®^ 480 (Roche Diagnostics International Ltd., Rotkreuz, Switzerland). 15% of the DNA samples used were randomly regenotyped for an accuracy test. The repeatability of the results was complete (100%). An expression quantitative trait loci (eQTL) analysis was conducted to determine whether the studied variants affect gene expression “https://gtexportal.org/home/” (accessed on 19 September 2024). The functional impact of the missense variant rs10467770 was predicted by three algorithms: SIFT (Sorting Intolerant from Tolerant) https://sift.bii.a-star.edu.sg/ (accessed on 20 September 2024), PolyPhen-2 (Polymorphism Phenotyping v2) http://genetics.bwh.harvard.edu/pph2/ (accessed on 19 September 2024), and CADD (Combined Annotation Dependent Depletion) https://cadd.bihealth.org/ (accessed on 19 September 2024) [14,15,16].

### 2.3. Statistical Analysis

Statistical analysis was performed using Statistica 13.0 software (TIBCO Software Inc., Palo Alto, CA, USA). The Shapiro–Wilk test was used to assess the normality of the distribution of quantitative data. Quantitative data with normal distribution were presented as means (±SD, standard deviation), and the Student’s *t*-test was used to compare dichotomous variables. Data with non-normal distribution were shown as medians (±QD, quartile deviation), and these variables were compared using the Mann–Whitney U test.

To check the possible association of the studied polymorphisms with ASD, the transmission disequilibrium test (TDT) was used. The TDT is based on an analysis of the transmission of specific alleles from heterozygous parents to their affected children. For this reason, only informative trios (families with at least one heterozygous parent) were included for the TDT analysis. The transmission of alleles was computed using a χ^2^ test as well as a Hardy–Weinberg equilibrium test and association analyses with traits like perinatal trauma, assessment of motor skills, communication skills, and observation of behavior. Risk ratios (RRs with 95% confidence intervals, CI) were computed using univariate analysis. Haplotype blocks were estimated using HaploView 4.2 [17] software. The algorithm of Gabriel et al. [18] was used for calculations of linkage disequilibrium (D′ and R^2^). Linkage disequilibrium for the CEU population was calculated using LDmatrix Tool [12]. The results were considered statistically significant at *p* < 0.050. In the case of multiple comparisons, the *p* values were adjusted using the Hochberg correction [19].

## 3. Results

The clinical characteristics of the study group are presented in Table 1. There were no statistically significant differences in the distribution of the characteristics presented in Table 1 between the sexes, except for the frequency of mobility/vitality. Among boys, the frequency of mobile/vital children was significantly higher than among girls (n = 119/146 boys, 81.51% vs. n = 27/43 girls, 62.79%, *p* = 0.010).

Table 2 shows the genotype distribution of the studied polymorphisms both in the group of children with ASD and their parents. The completeness of genotyping ranged between 85 and 90% depending on the polymorphism (Table 2). The genotype frequencies in the individual groups were similar (*p* > 0.050). The distribution of the alleles and genotypes of all analyzed polymorphisms was consistent with the Hardy–Weinberg equilibrium (*p* > 0.050).

**Figure 3:**
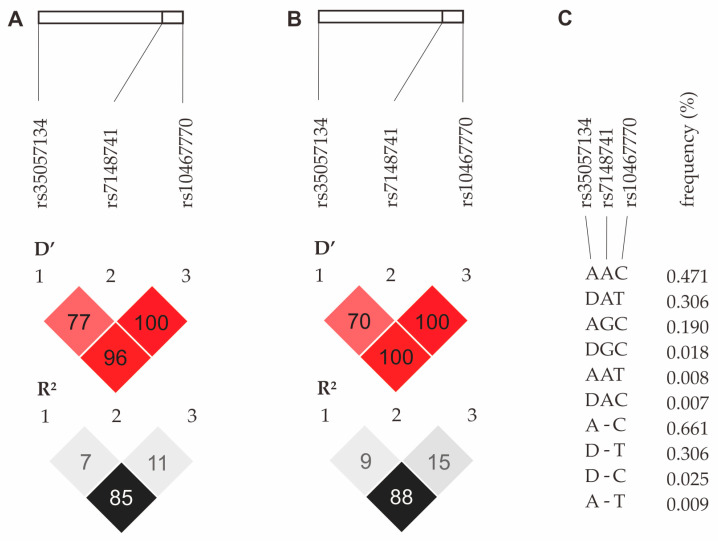
Haplotype analysis of the *CHD8* gene polymorphisms in the study group of children with ASD (**A**) and the CEU population (**B**). Frequencies of haplotypes in children with ASD (**C**). Legend: D, deletion allele; D’, relative linkage disequilibrium; R^2^, square of correlation coefficient between pairs of loci.

In the analyzed group of children with autism spectrum disorder, the studied polymorphisms did not form haplotype blocks, despite the relatively high R^2^ value observed between the rs35057134 and rs10467770 polymorphisms (Figure 3A). These results are consistent with those for the CEU population (USA Utah residents with ancestry from northern and western Europe, GRCh38 High Coverage) (Figure 3B). The frequencies of SNP variants in the group of children with ASD are shown below (Figure 3C).

### 3.1. Functionality Analysis of Studied Polymorphisms

**Figure 4:**
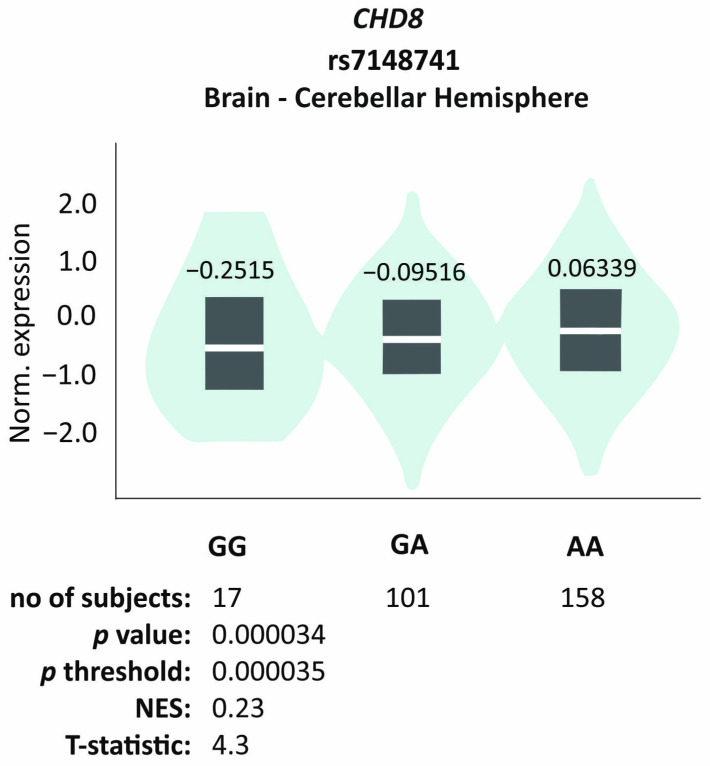
*CHD8* rs7148741 SNP impact on gene expression in the cerebellar hemisphere tissue. Based on GTEx portal.

The eQTL analysis revealed that only the rs7148741 variant affects gene expression in the cerebellar hemisphere tissue (*p* = 0.000034, NES = 0.23, T = 4.3; Figure 4). The functional impact of the missense variant rs10467770 was predicted by three algorithms: SIFT, PolyPhen-2, and CADD. This variant overlaps 14 transcripts, and for 2 of them only one algorithm predicted pathogenicity (i.e., probably damaging, SIFT score = 0.05, or deleterious, PolyPhen score = 0.987). Overall, it might suggest that the variant is more likely to be benign.

### 3.2. Allele Transmission in an Intrafamily Model

Complete families, including both biological parents and their child/children, were examined using the transmission/disequilibrium test. Our observations showed that the transmission frequency of all alleles of each analyzed polymorphism from parents to children was comparable (Table 3), and the results were independent of sex.

### 3.3. Association of CHD8 Gene Polymorphisms with Clinical Features of ASD

Further analyses included examining the association between specific *CHD8* gene polymorphisms and clinical features/symptoms of ASD, such as the newborn’s condition after birth, neurological and further motor development, speaking abilities, and the necessity of specialized care. In general, we demonstrated herein the association of all analyzed *CHD8* gene polymorphisms with the clinical phenotype of ASD.

### 3.4. The rs35057134 Polymorphism

We showed that the AA homozygotes of the rs35057134 polymorphism were more frequently predisposed to the use of an incubator, sleep disorders, heart rate fluctuations, and hospitalization (RR = 3.83, 95%CI: 1.46–10.10; RR = 1.40, 95%CI: 1.03–1.90; RR = 3.24, 95%CI: 1.21–8.70; RR = 1.29, 95%CI: 1.02–1.63, respectively). An analysis of the quantitative data indicated that the A allele carriers also showed a lower mean Apgar score compared to the DD homozygotes of rs35057134 (*p* = 0.009) (Table 4). It was also shown that the time spent in an incubator was the shortest in deletion homozygotes (DD), while the differences between D allele carriers and AA homozygotes were statistically significant (Table 4). There were no differences in birth weight between genotype variants of the rs35057134 polymorphism. The frequency of only a few parameters between genotypes of the rs35057134 polymorphism was statistically significant after correction for multiple comparisons (Table 4).

**Figure 5:**
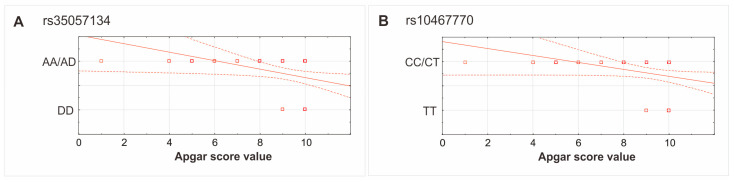
The distribution of Apgar score values with respect to rs35057134 (**A**) and rs10467770 (**B**) polymorphisms. Legend: D, deletion allele, squares—cases, Continuous line—regression line, Dashed line—confidence interval.

Additionally, the distribution of Apgar score values in carriers of the A allele revealed higher diversity (from 1 to 10 points) in the case of the DD homozygotes, amongst whom 10 or 9 points was mostly achieved (Figure 5A).

We also observed that the AA homozygotes appeared a higher risk of muscle hypotonia in the male subgroup (RR = 1.91, 95%CI: 1.11–3.28), however this association was not statistically significant after correction for multiple comparisons. This relationship was also not found in females (Table 5).

### 3.5. The rs10467770 Polymorphism

The CC homozygotes of the rs10467770 polymorphism were shown to predispose more frequently to the use of an incubator and hospitalization compared to the T allele carriers (RR = 3.35, 95%CI: 1.26–8.91; RR = 1.29, 95%CI: 1.02–1.64, respectively). Further statistical analysis showed the differences in mean Apgar score and duration of pregnancy among the C allele carriers and the TT homozygotes (*p* = 0.026 and *p* = 0.039, respectively) (Table 6). As in the case of the rs35057134 polymorphism, the distribution of Apgar score values between genotype variants of rs10467770 was different. In carriers of the C allele, it showed higher diversity (from 1 to 10 points) in comparison to the TT homozygotes (Figure 5B). There were no differences in birth weight or time of incubator use between genotype variants of the rs10467770 polymorphism. The frequency of only a few parameters between genotypes was statistically significant after correction for multiple comparisons (Table 6).

### 3.6. The rs7148741 Polymorphism

In the case of the rs7148741 polymorphism, we showed that the G allele carriers were predisposed to increased incidence of muscle hypotonia as compared to the AA homozygotes (RR = 1.70, 95%CI: 1.10–2.62). Moreover, the G allele carriers revealed lower birth weights in comparison to AA homozygotes (*p* = 0.038) (Table 7). There were no differences in the Apgar score results between genotype variants of the rs7148741 polymorphism, both in the additive and recessive/dominant models. Only the association between the G allele carrier state and muscle hypotonia (recessive/dominant model) showed statistical significance after taking into account the correction for multiple comparisons.

## 4. Discussion

In the current study, we analyzed the influence of *CHD8* gene polymorphisms on the occurrence and clinical phenotype of autism spectrum disorders. The analysis of allele transmission did not identify alleles that would be preferentially transmitted to affected children; however, we found an association of all selected *CHD8* gene polymorphisms, rs35057134, rs10467770 and rs7148741, with individual characteristics of the clinical phenotype of ASD. Carrying the G allele of rs7148741 predisposed an individual to muscle hypotonia and lower birth weight compared to the AA homozygotes; however, only the association with muscle hypotonia was statistically significant after correction for multiple comparisons. The AA homozygosity of the rs35057134 variant was associated with hypotonia (in the male subgroup), necessity of using of an incubator, sleep disorders, heart rate fluctuations, and hospitalization, in contrast to a D allele carrier. After correction for multiple comparisons, statistically significant differences remained in the case of necessity of using an incubator, heart rate fluctuations, and hospitalization. We also observed an association between the CC homozygosity of the rs10467770 variant and the use of an incubator, as well as hospitalization. Both these associations were significant after correction for multiple comparisons. Moreover, there were differences between C allele carriers and TT homozygotes (rs10467770) and between A allele carriers and DD homozygotes (rs35057134) in the mean Apgar score, both in univariate analysis and after Hochberg correction.

The *CHD8* gene regulates the expression of several thousand genes involved in nervous system development, extracellular matrix formation, and skeletal development [20], so its impact on the ASD phenotype may be multifaceted. High heterogeneity of the *CHD8* gene is considered one of the factors that may predispose an individual to the manifestation of the ASD phenotype. It is involved in the processes related to chromatin remodeling and fulfills various roles encompassing multiple biological pathways. A recent cohort study [21] showed that pathogenic variants of *CHD8* contribute to a broad range of phenotypic abnormalities, such as intellectual disability (68%), muscle hypotonia (29%), and a wide array of behavioral disorders (88%). Furthermore, abnormalities within the gastrointestinal system (53%), musculoskeletal system (79%), and urogenital system (18%) have been observed [22]. The involvement of *CHD8* variants in the manifestation of muscle hypotonia has also been confirmed by previous cohort studies [23]. Currently, it is challenging to definitively clarify whether the discussed muscle hypotonia is caused directly by a change in the expression of the *CHD8* gene or by a dysfunction of neighboring genes with its involvement [24]. Understanding this relationship precisely seems crucial, as considering different age groups of patients, muscle hypotonia was more frequently detected in the younger group, which may indicate it as a useful marker for early ASD diagnosis [25]. In order to explain the observed relationships between polymorphisms and clinical features, an in silico analysis of the potential impact of the tested variants on gene expression and protein function was conducted. The eQTL analysis revealed that only the rs7148741 variant affects gene expression in the cerebellar hemisphere tissue. For the missense variant rs10467770, some algorithms predicted a deleterious effect, though the overall analysis suggests it is likely benign.

This is the first study to indicate that the *CHD8* gene polymorphisms may affect the duration of incubator use. Previous research indicates differences in the development of premature newborns dependent on the place in the neonatal intensive care unit and, consequently, the stimulation of their development. Infants staying in private, single rooms with a limited number of parental visits were characterized by a reduction in normal hemispherical sulcal depth asymmetry and showed weaker development of language skills and motor functions. However, infants raised in open wards, having more frequent contact with the human voice and other stimulating factors, developed better [26]. Thus, it could be assumed that a long-term stay of a newborn in an incubator and limited sensory exposure may have negative effects on development, hence increasing the risk of ASD [27], in particular in children with genetic predispositions. In this context, this is also the first study to point out that neurodevelopmental disorders associated with *CHD8* gene polymorphisms may occur dependently on the period of time an incubator is used.

Additionally, genotypic variants of rs35057134 may determine sleep disorders in patients with ASD. The available data indicate the importance of the *CHD8* gene in maintaining circadian rhythms, which are needed for normal sleep cycles [28]. Mutations in the *CHD8*/*CHD7* chromatin remodeling gene may affect circadian rhythm disorders, as confirmed by case studies of children with ASD and studies in animal models [29,30]. In summary, it has been confirmed to date that sleep disorders accompanying ASD are complex and may result from the genetic, neurological, and behavioral backgrounds [31].

Notably, the current study is the first one carried out in a Caucasian population. Therefore, our results represent valuable information for a better understanding of the genetic basis of the autism spectrum, as well as the clinical features observed in this disorder. This seems particularly important in the context of the limited research on the impact of *CHD8* genotype variants on the clinical phenotype of ASD. It should be noted, however, that further studies are necessary to confirm the presented results and determine the pathomechanism of the identified associations.

### Limitations

A limitation of our study is the lack of detailed information regarding the functional role of the investigated polymorphisms. It should be, however, emphasized that the current work represents the first analysis in a Caucasian population in the context of ASD. Additionally, the relatively small sample size may have influenced the obtained results, although the observed association of all analyzed polymorphisms with the ASD phenotype does not appear to be random, as some remained significant even after correction for multiple comparisons. It is a controversial issue as to which set of hypotheses to treat as a family, that is, as the set for which correction is calculated [32]. We decided to treat the set as one family, as hypotheses about the lack of relationship between a specific genotype and phenotypic features in a specific group of subjects (23 tests), as applying the correction to all tests performed in this work could increase the probability of false negatives. We realize that our approach may be controversial, but multiple testing is a complex issue without a simple solution, and opinions among scientists differ. Nevertheless, further basic research is necessary to better understand the role of the *CHD8* gene in the etiology of ASD and its phenotype.

## 5. Conclusions

In summary, this study appears to confirm the hypothesis that the *CHD8* polymorphisms determine the phenotype of ASD, particularly in the context of muscle hypotonia (rs7148741), use of an incubator and hospitalization (rs35057134 and rs10467770), heart rate fluctuations (rs35057134), and median Apgar score (rs10467770 and rs35057134). The results we present may indicate the direction of future research and contribute to the selection of diagnostic markers for ASD.

## Figures and Tables

**Table 1:** Clinical characteristics of the study group.

Characteristics (n of Subjects Analyzed for Parameter)	ASD Children	Males	Females
	mean ± SD	mean ± SD	mean ± SD
- age (n = 209), [months]	87.08 ± 32.75	89.05 ± 32.62	79.89 ± 32.56 *
- birth weight (n = 204), [kg]	3.37 ± 0.61	3.42 ± 0.60	3.22 ± 0.59 *
- duration of pregnancy (n = 200), [weeks]	38.70 ± 2.17	38.71 ± 2.14	38.67 ± 2.32
- Apgar score (n = 201), [points]	9.27 ± 1.40	9.24 ± 1.46	9.39 ± 1.17
- time of incubator use (n = 196), [h]	28.57 ± 184.67	21.56 ± 106.77	55.07 ± 348.50
	n (%)	n (%)	n (%)
- sex (n = 210)	210 (100)	165 (78.64)	45 (21.36)
- perinatal trauma (n = 206)	9 (4.37)	6 (2.91)	3 (1.46)
- excessively calm infant (n = 189)	34 (17.99)	24 (12.70)	10 (5.29)
- restless infant (n = 189)	76 (40.21)	63 (33.33)	13 (6.88)
- abnormal motor development (n = 193)	82 (39.80)	64 (33.16)	18 (9.33)
- muscle hypertonia (n = 196)	38 (19.39)	33 (16.84)	5 (2.55)
- muscle hypotonia (n = 193)	69 (35.75)	51 (26.42)	18 (9.33)
- regression in communication (n = 198)	79 (39.90)	65 (32.83)	14 (7.07)
- impairment in eye contact (n = 199)	158 (79.40)	122 (61.31)	36 (18.09)
- compulsive, ritualistic behavior (n = 191)	143 (74.87)	112 (58.64)	31 (16.23)
- self-aggressive behavior (n = 199)	80 (40.20)	59 (29.65)	21 (10.55)
- hearing impairments (n = 197)	32 (16.24)	25 (12.69)	7 (3.55)
- vision impairments (n = 195)	54 (27.69)	45 (23.08)	9 (4.61)
- sleep impairments (n = 200)	97 (48.50)	71 (35.50)	26 (13.00)
- falling asleep impairments (n = 195)	60 (30.77)	45 (23.08)	15 (7.69)
- mobility/vitality (n = 189)	146 (77.25)	119 (62.96)	27 (14.29) *

* *p* < 0.050.

**Table 2:** Distribution of genotypes and alleles in children and their biological parents.

Genotype	Genotype Distribution, n (%)		
	Children	Mothers	Fathers
rs7148741 n	180	179	178
AA	111 (61.67)	117 (65.36)	120 (67.42)
AG	62 (34.44)	53 (29.61)	52 (29.21)
GG	7 (3.89)	9 (5.03)	6 (3.37)
A	284 (78.89)	287 (80.17)	292 (80.22)
G	76 (21.11)	71 (19.83)	64 (19.78)
rs35057134 n	183	182	183
AA	81 (44.26)	78 (42.86)	77 (42.08)
AD	83 (45.35)	77 (42.31)	86 (48.63)
DD	19 (10.39)	27 (14.83)	20 (9.29)
A	245 (66.94)	233 (64.01)	240 (65.57
D	121 (33.06)	131 (35.99)	126 (34.43)
rs10467770 n	181	183	183
CC	83 (45.86)	85 (46.45)	86 (46.99)
CT	82 (45.30)	72 (39.34)	77 (42.08)
TT	16 (8.84)	26 (14.21)	20 (10.93)
C	248 (68.51)	242 (66.12)	249 (68.03)
T	114 (31.49)	124 (33.88)	117 (31.97)

Legend: D, deletion allele.

**Table 3:** Transmission/disequilibrium test results.

SNP	Allele	Transmitted n (%)	Not Transmitted n (%)	χ^2^; *p*
rs7148741 *	A	45 (44.12)	57 (55.88)	1.41; 0.235
	G	57 (55.88)	45 (44.12)	1.41; 0.235
rs35057134 **	A	85 (54.49)	71 (45.51)	1.26; 0.262
	D	71 (45.51)	85 (54.49)	1.26; 0.262
rs10467770 ***	C	77 (53.47)	67 (46.53)	0.69; 0.405
	T	67 (46.53)	77 (53.47)	0.69; 0.405

* n of informative trios = 93. ** n of informative trios = 125. *** n of informative trios = 120.

**Table 4:** Distribution of the rs35057134 genotypes dependently on the presence/absence of ASD clinical phenotypes.

Characteristics (n of Subjects Analyzed for Parameter)		Genotypes						*p*	
		AA		AD		DD		Additive Model	Recessive/Dominant AA vs. AD/DD
		n	%	n	%	n	%		
The need to use an incubator (n = 180)	Yes	15	75.00	5	25.00	0	0.00	0.008 ^§^	0.003 ^1,^*^,§^
	No	64	40.00	77	48.13	19	11.88		
Sleep disorders (n = 174)	Yes	44	42.38	35	41.67	5	5.95	0.037	0.032 ^2^
	No	33	36.26	44	48.35	13	15.38		
Heart rate fluctuations (n = 173)	Yes	13	72.22	4	22.22	1	5.56	0.044	0.012 ^3,^*^,§^
	No	64	41.29	73	47.10	18	11.61		
Hospitalization (n = 150)	Yes	53	50.48	38	36.19	14	13.33	0.017 ^§^	0.034 ^4^
	No	22	33.85	38	58.46	5	7.69		
		Mean	±SD	Mean	±SD	Mean	±SD	AA/AD vs. DD	
Apgar score [points] (n = 176)		9.00	1.56	9.25	1.49	9.89	0.32	0.017 ^§^	0.009 ^§^
									AA vs. AD/DD
Incubator use [h] (n = 172)		33.90	137.28	34.41	255.11	0.00	0.00	0.035	0.033

Legend: D, deletion allele; *, with Yates’ exact; ^§^, differences significant after Hochberg correction for multiple comparisons. ^1^ RR = 3.83, 95%CI: 1.46–10.10. ^2^ RR = 1.39, 95%CI: 1.02–1.88. ^3^ RR = 3.24, 95%CI: 1.21–8.70. ^4^ RR = 1.29, 95%CI: 1.02–1.63.

**Table 5:** Distribution of the rs35057134 genotypes dependently on the presence/absence of muscle hypotonia in the male subgroup.

Sex (n of Subjects Analyzed for Parameter)	Muscle Hypotonia	Genotypes				χ^2^; *p*
		AA		AD/DD		Recessive/Dominant AA vs. AD/DD
		n	%	n	%	
Males (n = 133)	Yes	25	62.50	15	37.50	5.25; 0.022 ^1^
	No	38	40.86	55	59.14	
Females (n = 35)	Yes	5	41.67	10	58.33	0.07; 0.797
	No	7	43.48	13	56.52	

Legend: D, deletion allele. ^1^ RR = 1.91, 95%CI: 1.11–3.28.

**Table 6:** Distribution of the rs10467770 genotypes dependent on the presence/absence of ASD clinical phenotypes.

Characteristics (n of Subjects Analyzed for Parameter)		Genotypes						*p*	
		CC		CT		TT		Additive Model	Recessive/Dominant CC vs. CT/TT
		n	%	n	%	n	%		
The need to use an incubator (n = 178)	Yes	14	73.68	5	26.32	0	0.00	0.025	0.009 ^1,^*^,§^
	No	67	42.14	76	47.80	16	10.06		
Hospitalization (n = 169)	Yes	54	51.92	38	36.54	12	11.54	0.019 ^§^	0.036 ^2^
	No	23	35.38	38	58.46	4	6.15		
		Mean	±SD	Mean	±SD	Mean	±SD	CC/CT vs. TT	
Apgar score [points] (n = 178)		8.97	1.56	9.31	1.46	9.87	0.34	0.023	0.026 ^§^
Duration of pregnancy [weeks] (n = 175)		38.59	2.30	38.62	2.30	39.73	1.03	-	0.039

Legend: *, with Yates’ exact; ^§^, differences significant after Hochberg correction for multiple comparisons. ^1^ RR = 3.35, 95%CI: 1.26–8.91. ^2^ RR = 1.29, 95%CI: 1.02–1.64.

**Table 7:** Distribution of the rs7148741 genotypes dependent on the presence/absence of ASD clinical phenotypes.

Characteristics (n of Subjects Analyzed for Parameter)		Genotypes						*p*	
		AA		AG		GG		Additive Model	Recessive/Dominant AG/GG vs. AA
		n	%	n	%	n	%		
Muscle hypotonia	Yes	26	48.15	25	46.30	3	5.55	0.040	0.016 ^§^
(n = 165)	No	75	67.57	34	30.63	2	1.80		
		Mean	±SD	Mean	±SD	Mean	±SD	AG/GG vs. AA	
Birth weight [kg] (n = 175)		3.42	0.63	3.24	0.60	3.17	0.50	-	0.038

Legend: ^§^, difference significant after Hochberg correction for multiple comparisons.

## Data Availability

Dataset available on request from the authors.
